# Structural Correlation of Some Heterocyclic Chalcone Analogues and Evaluation of Their Antioxidant Potential

**DOI:** 10.3390/molecules181011996

**Published:** 2013-09-26

**Authors:** C. S. Chidan Kumar, Wan-Sin Loh, Chin Wei Ooi, Ching Kheng Quah, Hoong-Kun Fun

**Affiliations:** 1 X-ray Crystallography Unit, School of Physics, Universiti Sains Malaysia, Penang 11800 USM, Malaysia; E-Mails: chidankumar@gmail.com (C.S.C.K.); wansin_loh@live.com (W.-S.L.); ooichinwei88@gmail.com (C.W.O.); ckquah@usm.my (C.K.Q.); 2 Department of Chemistry, Alva’s Institute of Engineering & Technology, Mijar, Moodbidri 574225, India; 3 Department of Pharmaceutical Chemistry, College of Pharmacy, King Saud University, Riyadh 11451, Saudi Arabia

**Keywords:** heterocyclic, radical scavenging, reducing, substituent, overlay

## Abstract

A series of six novel heterocyclic chalcone analogues **4**(**a**–**f**) has been synthesized by condensing 2-acetyl-5-chlorothiophene with benzaldehyde derivatives in methanol at room temperature using a catalytic amount of sodium hydroxide. The newly synthesized compounds are characterized by IR, mass spectra, elemental analysis and melting point. Subsequently; the structures of these compounds were determined using single crystal X-ray diffraction. All the synthesized compounds were screened for their antioxidant potential by employing various *in vitro* models such as DPPH free radical scavenging assay, ABTS radical scavenging assay, ferric reducing antioxidant power and cupric ion reducing antioxidant capacity. Results reflect the structural impact on the antioxidant ability of the compounds. The IC_50_ values illustrate the mild to good antioxidant activities of the reported compounds. Among them, **4f** with a *p*-methoxy substituent was found to be more potent as antioxidant agent.

## 1. Introduction

Chalcones are important constituents of natural products. They are abundant in edible plants where they are considered to be the precursors of flavonoids and isoflavonoids. There is a growing interest in the pharmacological potential of chalcones, which constitute an important group of natural and synthetic products that have been screened for their wide range of pharmacological activities as antibacterial [1,2], anti-tumor [3,4,5], anti-inflammatory [6,7,8,9], antifungal [10] and antioxidant agents [11,12,13,14,15]. Chalcones are well known intermediates for synthesizing various heterocyclic compounds. Several methods have been reported for the synthesis of chalcones, among which the aldol condensation and Claisen–Schmidt condensation still occupy prominent positions. The Claisen-Schmidt condensation between aryl ketones and aromatic aldehydes in acidic or basic media gives chalcones. Chalcones are characterized by possessing an enone moiety between two aromatic rings. In an effort to diversify the pharmacological activities of conventional chalcones, a series of heterocyclic chalcone analogues in which an electron rich thiophene heterocycle replaces the benzene ring were synthesized.

Based on the above observations, herein we report the synthesis of some novel heterocyclic chalcone analogues using a conventional base-catalyzed Claisen-Schmidt condensation reaction. The structures of these compounds are extensively characterized by single crystal X-ray diffraction studies and evaluation of possible antioxidant activities has been carried out. 

## 2. Results and Discussion

**Scheme 1:**
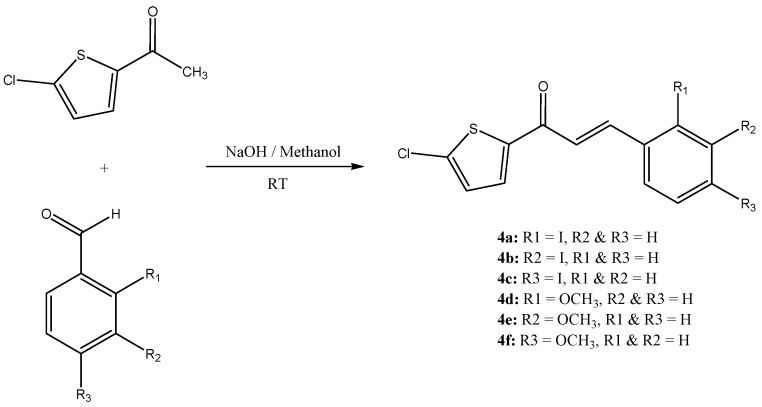
Synthesis of heterocyclic chalcone analogues.

Chalcones, a versatile class of natural and synthetic compounds, attract researchers for their immense range of biological activities. Chalcones containing thiophene moieties are supposed to further enhance the scope of these activities. The best known method employed for the synthesis of chalcones is the Claisen–Schmidt condensation between acetophenone and benzaldehyde in basic media. New chalcones have been designed and synthesized by the reaction of 2-acetyl-5-chlorothiophene with substituted benzaldehydes in presence of catalytic amount of NaOH in methanol, as shown in Scheme 1. The crystallographic data and parameters for structure refinement are listed in Table 1 and hydrogen bonding interactions are given in Table 2.

**Table 1:** Crystal data and parameters for structure refinement of compounds **4**(**a**-**f**).

Compound	4a	4b	4c	4d	4e	4f
CCDC deposition numbers	939878	942739	942740	948858	944070	944071
Molecular formula	C_13_H_8_ClIOS	C_13_H_8_ClIOS	C_13_H_8_ClIOS	C_14_H_11_ClO_2_S	C_14_H_11_ClO_2_S	C_14_H_11_ClO_2_S
Molecular weight	374.60	374.60	374.60	278.74	278.74	278.74
Crystal system	Monoclinic	Triclinic	Triclinic	Monoclinic	Monoclinic	Monoclinic
Space group	*Cc*	*P*-1	*P*-1	*P*2_1_	*P*2_1_/*c*	*Pc*
*a* (Å)	34.308(7)	6.0486(8)	6.1259(4)	11.9567(15)	5.9343(6)	3.9116(9)
*b* (Å)	4.1026(9)	8.4673(15)	8.6600(6)	17.986(2)	30.575(3)	15.867(4)
*c* (Å)	23.367(5)	13.5924(15)	13.5854(9)	12.2265(15)	7.3758(7)	10.146(2)
α (°)	90	88.616(2)	98.330(1)	90	90	90
β (°)	126.544(10)	86.511(2)	98.812(1)	90.01	104.623(3)	95.115(4)
γ (°)	90	70.131(2)	110.605(1)	90	90	90
*V* (Å^3^)	2642.4(9)	653.48(16)	651.06(8)	2629.3(5)	1294.9(2)	627.2(3)
*Z*	8	2	2	8	4	2
*D*_calc_ (g cm^−3^)	1.883	1.904	1.911	1.408	1.430	1.476
Crystal dimensions (mm)	0.35 × 0.17 × 0.09	0.52 × 0.22 × 0.09	0.76 × 0.41 × 0.13	0.69 × 0.15 × 0.06	0.56 × 0.40 × 0.07	0.66 × 0.26 × 0.07
Colour	Colourless	Colourless	Colourless	Yellow	Colourless	Colourless
μ (mm^−1^)	2.76	2.79	2.80	0.44	0.45	0.46
Radiation λ (Å)	0.71073	0.71073	0.71073	0.71073	0.71073	0.71073
*T*_min_/*T*_max_	0.448/0.779	0.322/0.779	0.223/0.705	0.753/0.972	0.790/0.971	0.753/0.968
Reflections measured	19059	10400	10227	19862	15699	6086
Ranges/indices (*h*, *k*, *l*)	−44, 44; −5, 5; −30, 30	−7, 7; −10, 10; −17, 17	−7, 7; −11, 11; −17, 16	−14, 14; −21, 20; −14, 14	−8, 8; −44, 42; −9, 10	−5, 4; −22, 22; −14, 14
θ limit (°)	2.4–29.1	1.5–26.5	1.6–27.5	2.8–26.1	1.3–31.2	2.6–39.9
Unique reflections	5911	2662	2972	8732	4170	3237
Observed reflections (*I* > 2σ(*I*))	4676	2482	2735	6388	2644	2846
Parameters	308	154	154	650	163	163
Goodness of fit on *F*^2^	1.09	1.13	1.08	0.97	1.02	1.04
*R*_1_, *wR*_2_ [*I* ≥ 2σ(*I*)]	0.068, 0.186	0.039, 0.127	0.036, 0.098	0.044, 0.113	0.047, 0.144	0.036, 0.086

**Table 2:** Hydrogen bond geometries for compounds **4d**, **4e** and **4f**.

*D*–H···*A*	*d*(*D*–H) (Å)	*d*(H···*A*)(Å)	*d*(*D*···*A*)(Å)	*Angle* (*D*–H···*A*)(°)
**4d**				
C3A—H3AA···O1D ^i^	0.93	2.45	3.319(6)	156
C3B—H3BA···O1C ^ii^	0.93	2.43	3.288(6)	154
C3C—H3CA···O1B ^iii^	0.93	2.42	3.277(6)	154
C3D—H3DA···O1A ^iv^	0.93	2.43	3.275(7)	151
**4e**	0.96	2.87	3.620(2)	136
C14-H14B··· *Cg*1				
**4f**				
C3-H3A···O1 ^v^	0.93	2.54	3.426(3)	160

Symmetry code: ^(i)^ −*x*, *y* + 1/2, −z + 1; ^(ii)^ –*x* + 1, *y* + 1/2, −*z*; ^(iii)^ –*x* + 1, *y* − 1/2, −*z* + 1; ^(iv)^ −*x*, *y* − 1/2, −*z*; ^(v)^*x* − 1, −*y* + 1, *z* − 1/2.

### 2.1. X-ray Crystal Structure Description for Compound **4a**, **4b** and **4c**

**Figure 1:**
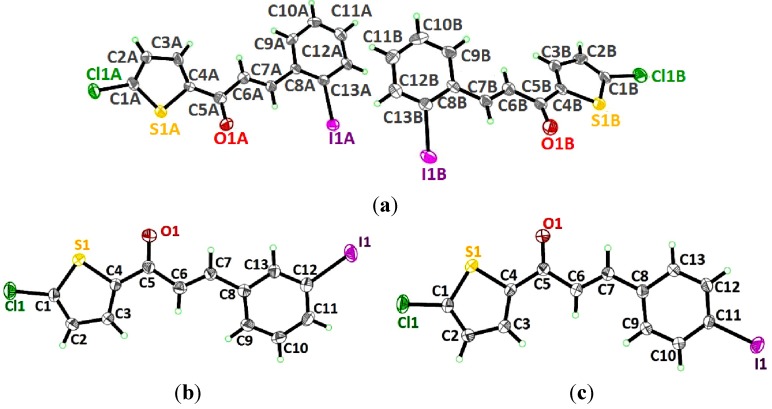
Molecular structures of compounds (**a**) **4a**, (**b**) **4b** and (**c**) **4c**, with atom numbering schemes. Displacement ellipsoids are drawn at the 30% probability level.

**Figure 2:**
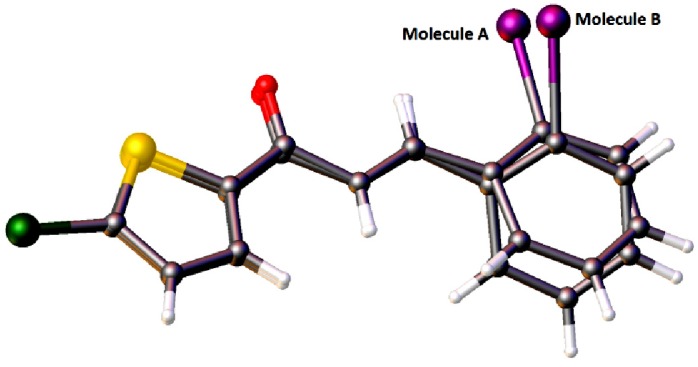
Overlay of all atoms in compound **4a**, calculated using the chlorothiophene moiety.

The molecular structures of **4a**, **4b** and **4c** are depicted in Figure 1a–c, showing the iodine substituents at the -*ortho* (**4a**), -*meta* (**4b**) and -*para* (**4c**) positions on the benzene rings. The asymmetric unit of compound **4a** contains two crystallographically independent molecules (molecules *A* and *B*, Figure 2). The compounds crystallized in the monoclinic, space group Cc (**4a**) and triclinic system, space group *P*1 (**4b** and **4c**). The three compounds exist in *E* configurations with respect to their C6=C7 double bonds with bond distances of 1.302 (18) Å for molecule *A* (1.334 (16) Å for molecule *B*) in **4a**, 1.313 (8) Å in **4b** and 1.331 (4) Å in **4c**. In all the three compounds, the molecules are almost planar, except for molecule *B* in **4a**. The essentially planar thiophene (S1/C1—C4) and the benzene (C8—C13) rings form dihedral angles of 5.3 (7)° for molecule *A* in **4a**, 10.8 (2)° in **4b** and 11.78 (15)° in **4c**, whereas these two rings are twisted away from each other for molecule B in **4a**, making a dihedral angle of 35.9 (7)°.

**Figure 3:**
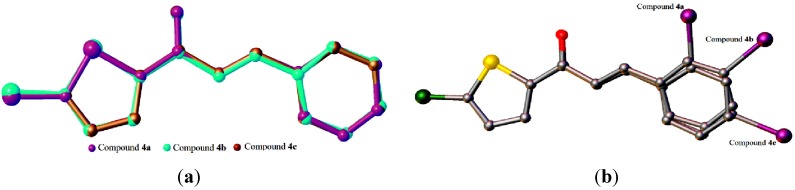
(**a**) Overlay of all non-H atoms, calculated using the chlorothiophene moiety. Molecule *B* in **4a** and the iodine substituents in all three compounds were excluded in the overlay. (**b**) Overlays were calculated using the chlorothiophene moiety in compounds **4a**, **4b** and **4c**. Hydrogen atoms have been omitted for clarity.

**Figure 4:**
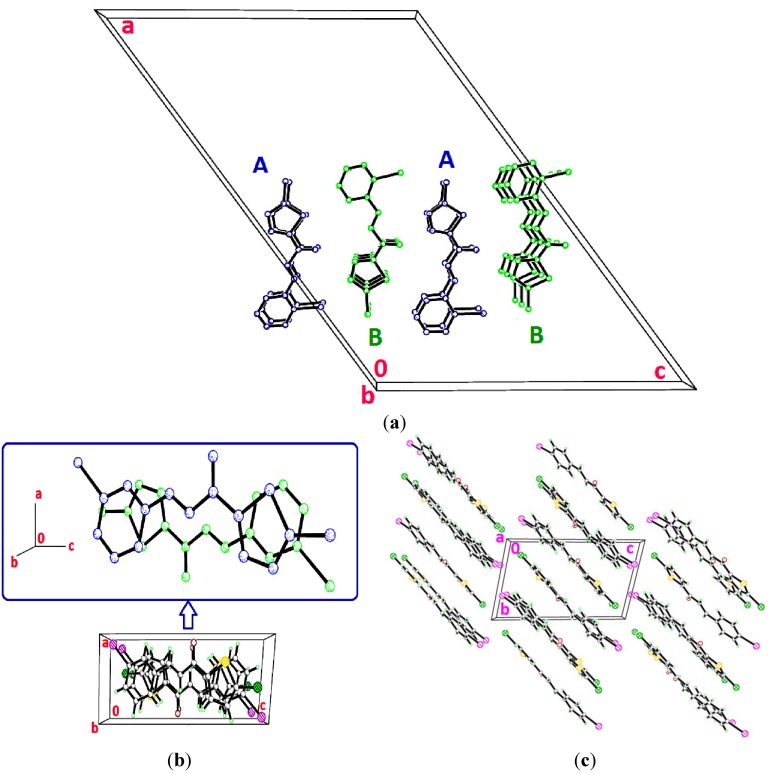
(**a**) Crystal structure of **4a** with molecule *A* represented in blue and molecule *B* in green, stacking along the *b*-axis. Hydrogen bonds have been omitted for clarity. (**b**) Packing diagram of 4b, showing the stacking in an inverse fashion. (**c**) Crystal structure of 4c, displaying the stacking of molecules along the (110) plane.

Molecules *A* and *B* in compound **4a** are overlaid over all the atoms as illustrated in Figure 2, with the r.m.s value of 0.426 Å. Figure 3a shows the overlays of all non-H atoms of **4a/4b**, **4a/4c** and **4b/4c**, calculated using the chlorothiophene moiety where their iodine substituents were excluded, with the r.m.s values of 0.064, 0.064 and 0.066 Å, respectively. The overlay including the iodine substituents is displayed in Figure 3b. Molecule A in compound **4a** is chosen as the overlay basis. The formation of short intra-molecular S···O contacts of 2.927(16) and 2.935(10) Å which is 0.39 and 0.38 Å shorter than the sum of van der Waals radii of the sulfur and oxygen atoms is observed in both the molecules A and B. This could be due to the charge delocalization into the carbonyl group from the thiophene ring which helps to stabilize the molecular structure. In the crystal packing of **4a**, as illustrated in Figure 4a, molecules *A*, which are represented in blue, and molecules *B* in green, stack one over the other along the *b*-axis, forming an inverted head-to-tail arrangement without any significant hydrogen bond interactions. The packing diagram of **4b** and **4c** are shown as Figure 4b,c, respectively. The molecules in **4b** and **4c** are stacked on each other in an inverse fashion. It is clearly shown in Figure 4b where the blue molecules are stacked inversely on the green molecule along the *b*-axis. Similarly, the molecules in **4c** are arranged in an inverse manner along the (110) plane. No classical hydrogen bond is found in both the crystal structures of **4b** and **4c**. 

### 2.2. X-ray Crystal Structure Description for Compounds **4d, 4e** and **4f**

**Figure 5:**
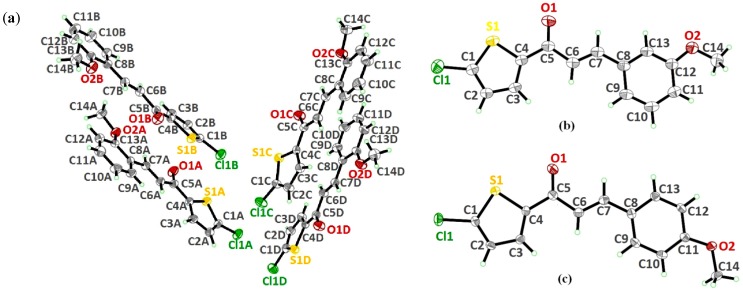
Molecular structure of compounds (**a**) **4d**, (**b**) **4e** and (**c**) **4f**, with atom numbering schemes. Displacement ellipsoids are drawn at the 30% probability level.

Figure 5a–c display the molecular structures of **4d**, **4e** and **4f**, which differ from each other in the -*ortho*, -*meta* and -*para* location of their methoxy substituents on the benzene rings. The compounds crystallized in the monoclinic system, space group *P*2_1_ (**4d**), *P*2_1_/*c* (**4e**) and *Pc* (**4f**), respectively. Unlike the other crystal structures, compound **4d** comprises four crystallography independent molecules (molecules *A*, *B*, *C* and *D*), in its asymmetric unit, with comparable geometrical parameters. The C=C double bonds of 1.318 (8), 1.317 (8), 1.338 (8) and 1.320 (8) Å with respect to the molecule *A*, *B*, *C*, *D* are *trans*-configured. The same configuration also exists in compound **4e** and **4f** with C=C double bonds of 1.319 (3) and 1.344 (3) Å. The molecules in these three compounds are almost planar as indicated by the dihedral angles of the two rings, thiophene (S1/C1—C4) and benzene rings (C8—C13), ranging from 2.11 (11) to 12.0 (3)° (Figure 5).

**Figure 6:**
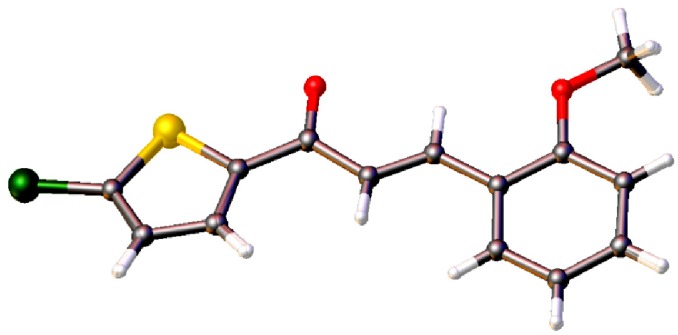
Overlay of all atoms in compound **4d**, comprising four independent molecules.

**Figure 7:**
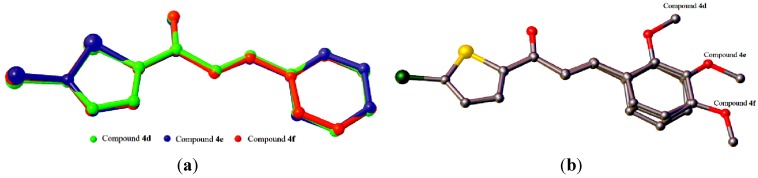
(**a**) Overlay of all non-H atoms, where the methoxy groups were excluded. (**b**) Overlays were calculated using the chlorothiophene moiety in compounds **4d**, **4e** and **4f**. Hydrogen atoms have been omitted for clarity. Only molecule *A* in compound **4d** is chosen for the overlay calculation.

**Figure 8:**
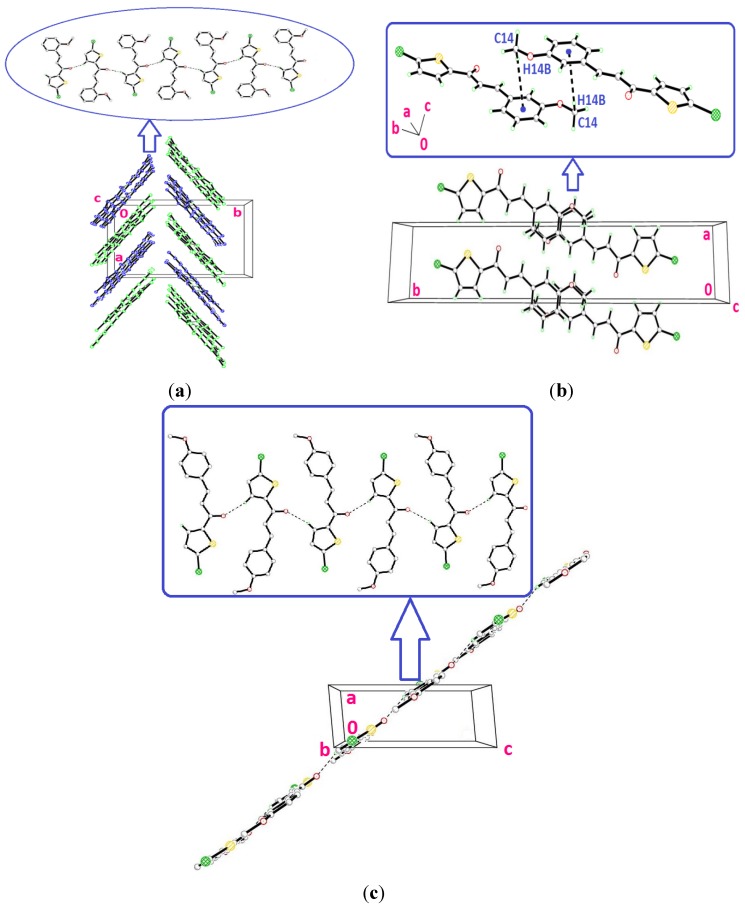
(**a**) Crystal structure of **4d** with the linkage of molecules *A* and *D* represented in blue and the linkage of molecules *B* and *C* in green, propagating along the *c*-axis. Hydrogen bonds not involved in the interactions have been omitted for clarity. Dashed lines are shown as intermolecular hydrogen bonds. (**b**) Packing diagram of **4e**, showing the C—H··· π interactions involving the centroids of the benzene ring. Dashed lines represent the C—H··· π interactions. (**c**) Crystal structure of **4f**, displaying the infinite chains running along the (201) plane. Intermolecular hydrogen bonds are represented as dashed lines.

The conformations of the four molecules in compound **4d** are very analogous, as shown in Figure 6, with the r.m.s value of 0.060 Å. Compounds **4d** (molecule A), **4e** and **4f** are overlaid over each other (Figure 7), giving the r.m.s value of **4d**/**4e** being 0.138 Å, of **4d**/**4f** being 0.104 Å and of **4e**/**4f** being 0.084 Å. As presented in Figure 8a, molecules A and D in **4d**, highlighted in blue, are linked together via intermolecular C3A—H3AA···O1D and C3D—H3DA···O1A hydrogen bonds (Table 2) into infinite chains with the A···D···A···D fashion. Likewise, the same pattern is adopted by molecules B and C where the intermolecular C3B—H3BA···O1C and C3C—H3CA···O1B hydrogen bonds (Table 2) link molecules B and C into another type of independent chains which are represented in green. These chains propagate along the *c*-axis. The crystal packing of **4e** is shown in Figure 8b, without the significant hydrogen bonds. The crystal structure stability in **4e** is provided by the C14—H14B··· π interactions (Table 2), involving the centroids of the benzene rings, whereas in **4f**, intermolecular C3—H3A···O1 hydrogen bonds (Table 2) bind the molecules to form infinite chains as depicted in Figure 8c, running along the (201) plane.

### 2.3. Antioxidant Activity

The synthesized compounds were screened for their antioxidant potential by employing the *in-vitro* assays such as DPPH free radical scavenging assay, ABTS radical scavenging assay, ferric ion reducing antioxidant power (FRAP) assay and cupric ion reducing antioxidant capacity (CUPRAC). 

#### 2.3.1. DPPH Radical Scavenging Assay

**Figure 9:**
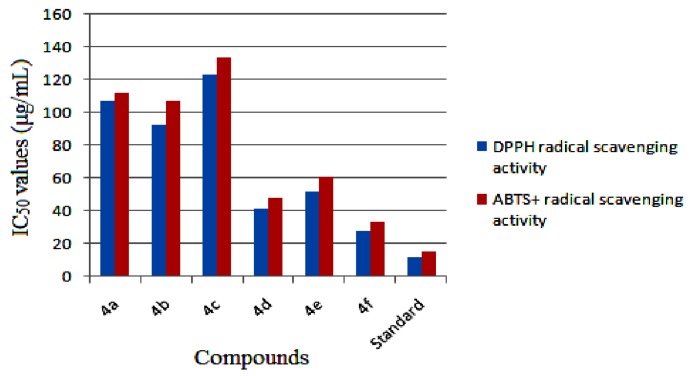
IC_50_ (concentration required for 50% inhibition) values for DPPH and ABTS^+^ radical scavenging activities of the compounds **4(a**–**f)** in comparison with the standard antioxidant BHT.

The DPPH radical scavenging test is a standard and widely used assay for *in vitro* antioxidant capacity to trap free radicals. The results of *in vitro* antioxidant activity (IC_50_ values) of the synthesized compounds in comparison with the reference antioxidant BHT are depicted in Figure 9. From the results, it is noticed that the compounds exhibit mild to good antioxidant activity. At the start with concentrations of 10–20 μg/mL, no significant change in the radical scavenging was noticed. However, the radical scavenging ability increased with increasing concentration of the samples (30–50 μg/mL). The IC_50_ values indicate that the chalcones **4d**, **4e** and **4f** showed good free radical scavenging activity. Among those, compound **4f** with a *p*-methoxy substituent was more potent in free radical scavenging compared to the other test compounds. The compounds **4**(**a**–**c**) bearing electronegative iodine substitution displayed mild scavenging activity. Among them, compound **4c** exhibited least activity.

#### 2.3.2. ABTS Radical Scavenging Assay

ABTS radical scavenging assay is a widely used method to evaluate the capacity of the test samples to trap free radicals. ABTS [2,2′-azino-bis(3-ethylbenzothiazoline-6-sulphonic acid)], when reacted produces the ABTS^+^ radical cation giving a green color in solution, for which the absorbance is measured at 730 nm. Antioxidants suppress this reaction by donating electron(s) and hinder the formation of the green colored ABTS^+^ radical cation. The amount of antioxidant in the test sample is thus inversely proportional to the formation of ABTS radical. The samples under test displayed mild to good ABTS radical scavenging activity and the results are comparable with DPPH radical scavenging ability. Compounds **4**(**d**–**f**) bearing methoxy substituents on the phenyl ring exhibited good activity, with **4f** being dominant, whereas the compounds **4**(**a**–**c**) bearing an electronegative iodine substituent displayed mild activity. The IC_50_ values are compared with the standard and depicted in Figure 9.

#### 2.3.3. Ferric Reducing Antioxidant Power (FRAP) Assay

**Figure 10:**
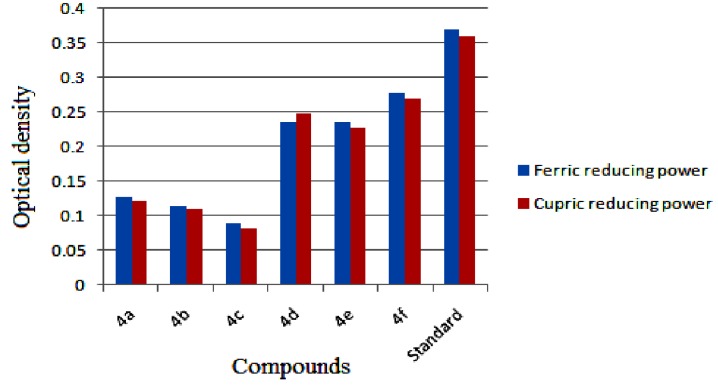
Comparison of ferric and cupric ion reducing ability of samples **4**(**a**–**f**) with reference to standard. Values are expressed as absorbance; high absorbance indicates high reducing power.

In practice, the antioxidant activity of a substance is directly correlated to its reducing ability. An assay like FRAP provides a reliable method to verify the antioxidant ability of a substance. Substances having a reduction potential react with potassium ferricyanide forming potassium ferrocyanide. The formed potassium ferrocyanide further reacts with FeCl_3_ to form an intensely colored Prussian blue complex which has a maximum absorbance at 700 nm. The amount of complex formed is directly proportional to the reducing capacity of the test sample. An increase in absorbance is a measure of the reducing power of the sample. Results are indicated in Figure 10. From the analysis it is clear that the compounds **4**(**d**–**f**) showed good ferric reducing ability, in which **4f** was the best compared to the other tested compounds and the compound **4c** showed the least reducing ability.

#### 2.3.4. Cupric Ion Reducing Antioxidant Capacity (CUPRAC) Assay

In this test the sample under evaluation effectively reduces Cu^2+^ to Cu^+^ changing the characteristic ion absorption. The reduced Cu^+^ ions combine with the chromogenic reagent neocuproine forming a stable 2:1 complex which has a maximum absorption at 450 nm. This method operates at pH 7. Results are shown in Figure 10, which indicates that the test compounds displayed a certain degree of reducing ability. The compound **4f** has a higher reducing ability than the other test compounds. The least reducing activity was shown by **4c**, which may be due to the electronegative iodine substituent at the *para* position on the aromatic ring.

## 3. Experimental

### 3.1. General Information

Melting points were determined on a Stuart Scientific (city, UK) apparatus. The purity of the compounds was confirmed by thin layer chromatography using Merck silica gel 60 F254 coated aluminium plates. The mass spectra were recorded on a Jeol JMS-D 300 mass spectrometer operating at 70 eV. Elemental analysis (CHN) was carried out on a Perkin Elmer Series II, 2400 analyzer. IR spectra were recorded as KBr pellets on a Perkin Elmer System 2000 FTIR spectrophotometer in the wave number range of 4,000–400 cm^−1^.

### 3.2. X-ray Crystallographic Analysis

X-ray analysis was done using Bruker SMART Apex II DUO CCDC diffractometer. The data were processed with SAINT and absorption correction was done using SADABS [16]. The structures were solved by direct method using the program SHELXTL [17], and were refined by full-matrix least squares technique on *F^2^* using anisotropic displacement parameters. The non-hydrogen atoms were refined anisotropically. In these compounds, all the H atoms were calculated geometrically with isotropic displacement parameters set to 1.2 (1.5 for methyl groups) times the equivalent isotropic *U* values of the parent carbon atoms. The overlay structures were drawn using Olex^2^ software [18]. The crystallographic data for the reported compounds are given in Table 1. H-bonding interactions are listed in Table 2. Crystallographic data have been deposited at the Cambridge Crystallographic Data Centre. CCDC No: 939878, 942739, 942740, 948858, 944070 and 944071 contain the supplementary crystallographic data for compounds **4**(**a**–**f**) respectively. Copies of the data can be obtained free of charge on application to the CCDC, 12 Union Road, Cambridge CB2 IEZ, UK. Fax: +44-(0)1223-336033 or E-Mail: deposit@ccdc.cam.ac.uk. 

### 3.3. General Procedure for the Synthesis of Chalcones **4(a–f)**

A mixture of 2-acetyl-5-chlorothiophene (0.01 mol) and substituted benzaldehyde (0.01 mol) was dissolved in methanol (20 mL). A catalytic amount of NaOH was added to the solution dropwise with vigorous stirring. The reaction mixture was stirred for about 3–4 h at room temperature. The resultant crude products were filtered, wash successively with distilled water and recrystallized from ethanol to get the corresponding chalcone. Crystals suitable for X-ray diffraction studies were obtained by the slow evaporation technique using a suitable solvent.

*(2E)-1-(5-Chlorothiophen-2-yl)-3-(2-iodophenyl)prop-2-en-1-one* (**4a**): Solvent for growing crystals: mixture of acetone, ethanol and acetonitrile (1:1:1 *v/v*); Yield: 64%; M.P.: 104–105 °C; IR (cm^−1^, KBr): 1645 (C=O), 1588 (HC=CH), 3079 (C-H), 806 (C-I), 795 (C-Cl, thiophene), 759 (C-S); LCMS: *m*/*z =* 375 (M^+^+1); Elemental analysis: Calculated for C_13_H_8_ClIOS: C, 41.68%; H, 2.15%; Found: C, 41.65%; H, 2.19%.

*(2E)-1-(5-Chlorothiophen-2-yl)-3-(3-iodophenyl)prop-2-en-1-one* (**4b**): Solvent for growing crystals: mixture of acetone, ethanol and acetonitrile (1:1:1 *v/v*); Yield: 67%; M.P.: 126–128 °C; IR (cm^−1^, KBr): 1644 (C=O), 1585 (HC=CH), 3077 (C-H), 802 (C-I), 794 (C-Cl, thiophene), 754 (C-S); LCMS: *m*/*z =* 375 (M^+^+1); Elemental analysis: Calculated for C_13_H_8_ClIOS: C, 41.68%; H, 2.15%; Found: C, 41.64%; H, 2.20%.

*(2E)-1-(5-Chlorothiophen-2-yl)-3-(4-iodophenyl)prop-2-en-1-one* (**4c**): Solvent for growing crystals: mixture of acetone, ethanol and acetonitrile (1:1:1 *v/v*); Yield: 66%; M.P.: 164–166 °C; IR (cm^−1^, KBr): 1647 (C=O), 1592 (HC=CH), 3078 (C-H), 805 (C-I), 791 (C-Cl, thiophene), 768 (C-S); LCMS: *m*/*z =* 375 (M^+^+1); Elemental analysis: Calculated for C_13_H_8_ClIOS: C, 41.68%; H, 2.15%; Found: C, 41.63%; H, 2.18%.

*(2E)-1-(5-Chlorothiophen-2-yl)-3-(2-methoxyphenyl)prop-2-en-1-one* (**4d**): Solvent for growing crystals: *N,N*-dimethylfomamide; Yield: 61%; M.P.: 134–135 °C; IR (cm^−1^, KBr): 1645 (C=O), 1579 (HC=CH), 3075 (C-H), 1226 (C-O-C), 795 (C-Cl, thiophene), 723 (C-S); LCMS: *m*/*z =* 279 (M^+^+1); Elemental analysis: Calculated for C_14_H_11_ClO_2_S: C, 60.32%; H, 3.98%; Found: C, 60.27%; H, 4.03%.

*(2E)-1-(5-Chlorothiophen-2-yl)-3-(3-methoxyphenyl)prop-2-en-1-one* (**4e**): Solvent for growing crystals: mixture of acetone, ethanol and acetonitrile (1:1:1 *v/v*); Yield: 69%; M.P.: 108–110 °C; IR (cm^−1^, KBr): 1646 (C=O), 1585 (HC=CH), 3072 (C-H), 1223 (C-O-C), 799 (C-Cl, thiophene), 723 (C-S); LCMS: *m*/*z =* 279 (M^+^+1); Elemental analysis: Calculated for C_14_H_11_ClO_2_S: C, 60.32%; H, 3.98%; Found: C, 60.29%; H, 4.01%.

*(2E)-1-(5-Chlorothiophen-2-yl)-3-(4-methoxyphenyl)prop-2-en-1-one* (**4f**): Solvent for growing crystals: mixture of acetone, ethanol and acetonitrile (1:1:1 *v/v*); Yield: 72%; M.P.: 118–119 °C; IR (cm^−1^, KBr): 1644 (C=O), 1586 (HC=CH), 3079 (C-H), 1227 (C-O-C), 798 (C-Cl, thiophene), 723 (C-S); LCMS: *m/z =* 279 (M^+^+1); Elemental analysis: Calculated for C_14_H_11_ClO_2_S: C, 60.32%; H, 3.98%; Found: C, 60.28%; H, 4.03%.

### 3.4. *In Vitro* Antioxidant Activity

#### 3.4.1. DPPH Radical Scavenging Assay

The ability of the test samples in addition to the standard antioxidant butylated hydroxytoluene (BHT) on DPPH radical scavenging was estimated according to the method described in the literature [19]. An aliquot (200 μL) of an ethanolic solution of the samples (0–50 μg/mL for samples; 0–5 μg/mL for BHT) was mixed with 100 mM tris-HCl buffer (800 μL, pH 7.4) and then 500 μM DPPH in methanol (1 mL) was added for a final concentration of 250 μM. The mixture was vigorously shaken and incubated in the dark at room temperature for 20 min. A DPPH blank solution (control) was prepared as above without the sample, and methanol was used for the baseline correction. The absorbance of the test solutions were measured spectrophotometrically at 517 nm. The DPPH radical scavenging activities were calculated using the equation:

DPPH radical scavenging activity (%) = [(Ac − As)/Ac] × 100
(1)

where Ac is the absorbance of the control and As is the absorbance of the test samples. The inhibition concentration of the samples for 50% (IC_50_) DPPH radical scavenging was also calculated. Results were expressed as mean of the three determinations.

#### 3.4.2. ABTS Radical Scavenging Assay

The antioxidant ability of the test compounds were evaluated in terms of the ABTS radical scavenging activity. The assay is performed according to the reported method [20]. ABTS^+^ was obtained by reacting 7 mM ABTS stock solution with 2.45 mM potassium persulfate and the mixture was incubated in dark at room temperature for about 12–16 h until the reaction was completed or the absorbance become stable. The solution was then diluted with 5 mM phosphate buffer (pH 7.4) to obtain an absorbance of 730 nm of 0.70 ± 0.02. One mL of the prepared ABTS^+^ solution was added to the different concentration of the test samples 4a–f (0–50 μg/mL) in ethanol and also to the control. After 30 min, the absorbance of the test samples was measured at 730 nm against the blank solution (without sample). The percentage ABTS^+^ scavenging is calculated by:

ABTS^+^ scavenging activity (%) = [(Ac − As)/Ac] × 100 (2)

where Ac is the absorbance of the control and As is the absorbance of the test samples. The inhibition concentration of the samples for 50% (IC_50_) ABTS^+^ radical scavenging was also calculated. Results were expressed as mean of the three determinations.

#### 3.4.3. Ferric Reducing Antioxidant Power (FRAP) Assay

The synthesized compounds were screened for ferric reducing power antioxidant ability by the method described by Oyaizu [21]. The method is based on the reduction of ferric (Fe^3+^) to ferrous (Fe^2+^), which is accomplished in the presence of antioxidants. Samples with different concentrations (0–50 μg/mL) were mixed with an equal volume of 0.2 M phosphate buffer (pH 6.6) and 1% potassium ferricyanide and the mixture were incubated for 20 min at 50 °C. The mixture was acidified with 10% trichloroacetic acid (2.5 mL) and then centrifuged at 3,000 rpm for about 15 min. The upper supernatant liquid was diluted with distilled water and 0.1% ferric chloride was added. The absorbance was measured at 700 nm. The increase in absorbance is directly proportional to the reducing ability of the compound. The blank solution was prepared as above without the sample.

#### 3.4.4. Cupric Ion Reducing Antioxidant Capacity (CUPRAC) Assay

Furthermore, the synthesized compounds were evaluated for their cupric ion reducing power by the reported method [22]. CUPRAC is a widely applicable method for evaluating the antioxidant properties of a substance. A mixture of CuCl_2_ (1 mL, 0.01 M) solution, ethanolic neocuproine (1 mL, 0.0075 M) and ammonium acetate (1 mL, 1 M) were dissolved and test samples (1 mL, 10 μM) were added along with distilled water (0.1 mL). The mixture was incubated for about 30 min and the absorbance was measured at 450 nm against the blank solution. Blank solution is prepared as above without the sample.

#### 3.4.5. Statistical Analysis

All the assay measurements were performed in triplicates (*n* = 3) and are expressed as mean of the three determinations. The amount of compound required to inhibit DPPH and ABTS free radicals by 50% (IC_50_) was graphically estimated using linear regression algorithm.

## 4. Conclusions

A series of new 5-chlorothiophene chalcones, **4**(**a**–**f**), were synthesized in good yield and characterized by IR, mass, elemental analysis and single crystal X-ray diffraction studies. In addition, their *in-vitro* antioxidant activity has been evaluated. The reported compounds displayed mild to good antioxidant properties, which is due to the presence of electronegative –*I* and electron donating -OCH_3_ substituent at different positions on the phenyl ring. The increasing order of antioxidant activity of the synthesized compounds follows the order **4f** > **4d** > **4e** > **4b** > **4a** > **4c**. The present study revealed that (1) the influence of the nature of the functional linkage (electron withdrawing and electron donating groups) and (2) the position of the substituent on the phenyl ring of 5-chlorothiophene chalcones are crucial for the exhibited antioxidant activities.
